# A Bayesian Approach for Evaluation of Determinants of Health System Efficiency Using Stochastic Frontier Analysis and Beta Regression

**DOI:** 10.1155/2016/2801081

**Published:** 2016-03-29

**Authors:** Talat Şenel, Mehmet Ali Cengiz

**Affiliations:** Department of Statistics, Ondokuz Mayıs University, 55139 Samsun, Turkey

## Abstract

In today's world, Public expenditures on health are one of the most important issues for governments. These increased expenditures are putting pressure on public budgets. Therefore, health policy makers have focused on the performance of their health systems and many countries have introduced reforms to improve the performance of their health systems. This study investigates the most important determinants of healthcare efficiency for OECD countries using second stage approach for Bayesian Stochastic Frontier Analysis (BSFA). There are two steps in this study. First we measure 29 OECD countries' healthcare efficiency by BSFA using the data from the OECD Health Database. At second stage, we expose the multiple relationships between the healthcare efficiency and characteristics of healthcare systems across OECD countries using Bayesian beta regression.

## 1. Introduction

Public expenditures on health are a matter of concern for governments in today's world. These expenditures have recently accelerated and are putting pressure on public budgets. Therefore, health policy makers have focused on the performance of their health systems and many countries have introduced reforms to improve the performance of their health systems. After two reports published by World Health Organization (WHO) which estimate health system efficiency for 191 countries between 1993 and 1997, studies on the health system efficiency have increased significantly [1, 2]. They used a panel-fixed effects model to create a production frontier with a time-invariant inefficiency term. They modelled health outcome as the output of the production function and health expenditures and their square and educational levels as the inputs. Hollingsworth and Wildman [3] and Greene [4] used the same variables with additional variables to estimate health system efficiency.

Stochastic Frontier Analysis (SFA) can be used for efficiency comparisons in the performance of health systems. Since WHO reports in 2000, SFA, which is a parametric approach, has been used only a few times in the literature to compare health system efficiency in OECD countries. In this study, one of our main targets is to use SFA with a Bayesian approach. In spite of many advantages, there are a few studies on SFA with a Bayesian approach. The Bayesian approach for the SFA was first introduced by van den Broeck et al. [5]. Koop et al. [6] describe the use of the Markov Chain Monte Carlo (MCMC) as a numeric integration method in a stochastic frontier framework. Also, [7–9] give current developments in BSFA. More recently, Griffin and Steel [10] describe MCMC methods for Bayesian analysis of stochastic frontier models using the WinBUGS package, freely available software. Tsionas and Papadakis [11] provide a Bayesian approach to the problem organized around simulation techniques. Tabak and Langsch Tecles [12] use a Bayesian stochastic frontier for cost and profit efficiencies of the Indian banking sector.

In recent literature, there are some more studies investigating the relationship between health spending and health outcomes. The stochastic frontier allows for the estimation of a potential level of outcome given expenditures in a country, where the deviation of the actual from the potential level is used in the computation of an inefficiency score. Wranik [13] assesses which policy-relevant characteristics of a healthcare system contribute to health system efficiency measured by using the stochastic frontier approach. de Cos and Moral-Benito [14] investigate the most important determinants of healthcare efficiency for OECD countries. In their study, first, the countries' health efficiencies were estimated and ranked using alternative parametric and nonparametric indices. At the second stage they regress efficiency scores on a set of 20 indicators representing health system characteristics based on the database by Paris et al. [15]. They use Data Envelopment Analysis (DEA) and SFA for estimating health system efficiency. Later, Least Square and Bayesian model averaging were selected as regression method.

In many research areas, it is very common to encounter the fact that the dependent variable takes values in the standard unit interval (0,1) such as rates, proportions, percentages, and fractions. There are some drawbacks when the linear regression model is applied to this kind of dependent variable. The major drawback is the fitted values for dependent variable which exceed its lower and upper bounds. To overcome these drawbacks, one way is to transform the dependent variable to values on the real line. However, this approach has also its own obstacles. The major obstacle is that the model parameters cannot be interpreted in terms of the original dependent variable. Ferrari and Cribari-Neto [16] introduced a beta regression model which is based on the assumption that the dependent variable is beta-distributed. Despite the many benefits associated with Bayesian beta regression, there are a few studies such as [17–20].

As mentioned earlier, there are two main alternative approaches considered in the literature for efficiency estimations in the performance of health systems. These are DEA and SFA. In Bayesian approach, we need a specific functional form for the production frontier and a source of randomness in production. SFA approach includes both components. So we prefer SFA with Bayesian approach to DEA at the first step of this study. For the second stage, classic linear regression and Tobit-type approaches have been used widely in recent literature. Also in a few studies Bayesian model averaging has been used recently. We prefer Bayesian beta regression for the benefits of robustness, since the other regression approach will produce more biased estimations.

In this study, the first aim is to estimate health system efficiency across OECD countries using BSFA. The second aim is to select the factors affecting the health system efficiency using Bayesian beta regression. We used the extended data set used by de Cos and Moral-Benito [14].

## 2. Materials and Methods

### 2.1. Bayesian Stochastic Frontier Model

SFA approach proposed by Aigner et al. [21] can be specified as follows:

(1) $$\begin{array}{c} y_{i}=X_{i}\beta +\varepsilon_{i},\;\varepsilon_{i}=v_{i}-u_{i},\;\;u_{i}\geq 0, \end{array}$$

where *y*_*i*_ is log output, *X*_*i*_ is a vector of input measures, *β* is the vector of coefficients, *v*_*i*_ is independent and identically distributed error term, and *u*_*i*_ ≥ 0 is technical inefficiency. Error term *ε*_*i*_ = *v*_*i*_ − *u*_*i*_ has a symmetric distribution.

The posterior distribution is shown as follows:

(2) Pβ,σ2,u,θYαPYX,β,σ2,u,θ·∏i=1NPuiθPβPσ2Pθ,

where *β* is the vector of coefficients, *θ* is the vector of parameters in the prior distribution of *u*_*i*_, and *X* is the matrix of the inputs.

### 2.2. Beta Regression Model

Let *y* be continuous variable which takes values in unit interval (0,1). The variable is assumed to be beta-distributed with the following parameterization:

(3) fy;μ,φ=ΓφΓμφΓ1−μφyμφ−11−y1−μφ−1,0<y<1,

where 0 < *μ* < 1 and *φ* > 0. Practitioners usually try to model this kind of variables such as proportions, percentages, or rates with regression analysis [22]. Beta regression is used when the response variable is restricted on the interval (0,1). Regression model is obtained with the parameterization of the mean and precision as *E*(*y*) = *μ* and Var(*y*) = *μ*(1 − *μ*)/(1 + *φ*).

The mean response is related to linear predictors through a monotonic and twice differentiable link function such that

(4) $$\begin{array}{c} g\left(\;\mu_{i}\;\right)=\overset{p}{\underset{k=1}{\sum }}x_{ik}\beta_{k}, \end{array}$$

where *β* = (*β*_1_, *β*_2_,…, *β*_*p*_) represents the beta coefficients on regression parameters, *x* = (*x*_1_, *x*_2_,…, *x*_*p*_) denotes the predictor variables in mean model, and *g*(·) shows the link function. It is possible to choose link functions between several functional forms such as logit, probit, and complimentary log-log. We get the logit form for the regression analysis. Beta regression model also contains a precision parameter *φ* for modelling the dispersion parameter which is the reciprocal of *φ*. For this study, we assumed the precision parameter as constant which does not vary across all observations.

Beta coefficients are estimated with the log-likelihood function of the model. Log-likelihood function of the model is

(5) ltμt,φ=log⁡Γφ−log⁡Γμtφ−log⁡Γ1−μtφ+μtφ−1log⁡yt+1−μtφ−1log⁡1−yt.

We can estimate the beta coefficients based for mean model on scoring functions derived from the log-likelihood function with numerical optimization methods. Most common used methods are Fisher scoring or Newton-Raphson algorithms for the maximum likelihood estimation of the model [23].

## 3. Results and Discussion

Our sample covers 29 OECD countries. The variables used in the first step for the estimation of efficiency indices are mainly taken from the OECD Health Database and cover the period between 1997 and 2009; in contrast, the variables referring to health system characteristics are obtained from Paris et al. [15] and represent a cross section for the year 2009. The measurement of productive efficiency is based on the relationship between output produced and inputs required for production. In this paper we consider life expectancy as output in the same way as [13, 24]. Turning to the inputs in the health production function, we consider per capita GDP, per capita health expenditures, education, tobacco consumption, alcohol consumption, fruit and vegetables consumption, and nitrogen oxide emissions.

The information referring to health system characteristics draws on a survey in which health authorities of each country respond to 269 questions on their healthcare system. Paris et al. [15] then summarise the information in 20 healthcare policy indicators that take values between 0 (minimum) and 6 (maximum). These indicators include information on the influence of the market and regulations on healthcare users, insurers and suppliers, the characteristics of basic healthcare coverage, the management of the healthcare budget, and the decision-making process in the provision of healthcare systems. de Cos and Moral-Benito [14] give a brief description of each of the 20 indicators. Those variables are choice of insurer (*X*_1_), insurer level for competition (*X*_2_), over the basic coverage (*X*_3_), degree of private provision (*X*_4_), volume incentives (*X*_5_), regulation of prices billed by providers (*X*_6_), user information on quality and prices (*X*_7_), regulation of the workforce and equipment (*X*_8_), patient choice among providers (*X*_9_), gate keeping (*X*_10_), price signals on users (*X*_11_), priority setting (*X*_12_), stringency of the budget constraint (*X*_13_), regulation of prices paid by third-party payers (*X*_14_), degree of decentralization (*X*_15_), degree of delegation to insurers (*X*_16_), consistency in responsibility (*X*_17_), breadth (*X*_18_), scope of basic coverage (*X*_19_), and depth of coverage (*X*_20_).

The proposed model at first stage is specified as follows:

(6) Yit=β0+β1GDPit+β2HEXit+β3EDUit+β4TOCit+β5ALCit+β6VECit+β7NITit+β8t+vit−uit,

where, for *i*th country in *t*th year, *Y*_*it*_ is the natural logarithm of life expectancy, GDP_*it*_ is the natural logarithm of per capita GDP, HEX_*it*_ is the natural logarithm of the per capita health expenditures, EDU_*it*_ is the education, TOC_*it*_ is the tobacco consumption, ALC_*it*_ is alcohol consumption, VEC_*it*_ is fruit and vegetables consumption, NIT_*it*_ is nitrogen oxide emissions, and *t* is a time trend. *v*_*it*_ is a symmetric disturbance capturing the effect of noise and as usual is assumed to be normal and *u*_*it*_ is interpreted as an indicator of the inefficient use of the life expectancy and is assumed to be a half-normal distribution, $u_{i}\overset{i.i.d.}{\sim }N^{+}(0,\lambda )$.

For Bayesian inference we need to specify prior distribution for all unknown parameters. All parameters are assumed to be a priori independent. We assign *β*_*j*_ ~ *N*(0, *σ*_*β*_^2^). For a half-normal distribution, we assign a Gamma prior for the precision, that is, *λ* ^−1^ ~ Ga(*c*, *d*). The complexity of these models makes it necessary to use numerical integration methods such as MCMC and in particular the Gibbs sampling algorithm with data augmentation as introduced by Koop et al. [6]. For the proposed model, implementation was carried out using the WinBUGS package.

Posterior summaries and densities for the proposed model after running the MCMC algorithm for 37500 iterations and discarding the initial 12000 are provided in Table 1. Table 1 shows posterior mean, sample standard deviation (SD), Monte Carlo error (MC error), and median with a 95% posterior credible interval. One way to assess the accuracy of the posterior estimates is by calculating the MC error for each parameter. This is an estimate of the difference between the mean of the sampled values and the true posterior mean. As a rule of thumb, the simulation should be run until the MC error for each parameter of interest is less than about 5% of the sample standard deviation. As seen in Table 1, the Monte Carlo error for each parameter of interest is less than about 5% of SD.

Table 1 shows that GPD, health expenditure, tobacco consumption, fruit and vegetables consumption, nitrogen oxide emissions, and time are significant, whereas the other parameters are not significant.

29 OECD countries' health system efficiencies based development performances are measured by using BSFA. Technical efficiency scores of the countries are estimated and, according to these scores, ranking of health system efficiency was presented in Table 2.

Based on the technical efficiency, while the most effective country was Australia, the lowest effective country was Turkey.

At second stage, Bayesian beta regression was performed to obtain the related factors with efficiency scores. The beta regression model used is as follows:

(7) $$\begin{array}{c} g\left(\;\mu_{i}\;\right)=\beta_{0}+\overset{20}{\underset{k=1}{\sum }}x_{ik}\beta_{k}, \end{array}$$

where *β* = (*β*_1_, *β*_2_,…, *β*_20_) represents the regression coefficients on regression parameters, *x* = (*x*_1_, *x*_2_,…, *x*_20_) denotes 20 healthcare policy indicators as the predictor variables, and *g*(·) shows the link function. We prefer the logit link for the sake of simplicity. In this regression model, 20 healthcare policy indicators described above are considered as independent variables and efficiency scores obtained from BSFA are considered as dependent variables. These efficiency scores are assumed to be beta-distributed.

For Bayesian inference, we need to specify prior distribution for all regression parameters. All parameters are assumed to be a priori independent. We assign all *β* ~ *N*(0, *σ*_*β*_^2^). Posterior summaries and densities for the Bayesian beta regression model after running the MCMC algorithm for 48000 iterations and discarding the initial 9500 are provided in Table 3. The posterior mean, SD, and MC error with a 95% posterior credible interval were presented in Table 3.

As seen in Table 3, the MC error for each parameter of interest is less than about 5% of SD. So convergence is satisfied for all parameters.

## 4. Conclusion

In this study, we used a Bayesian approach for stochastic frontier model to determine the health system performance of OECD countries. We proposed a Bayesian estimator for a stochastic frontier model with errors in variables. The inefficiency term of the model followed a half-normal distribution. For the Bayesian approach the Gibbs sampling algorithm with data augmentation was performed. Before using the Bayesian approach results, the convergence of all parameters of each model was satisfied. Results from BSFA show that while GDP, health expenditure, tobacco consumption, fruit and vegetables consumption, nitrogen oxide emissions, and time were significant, the other parameters such as education and alcohol consumption are not significant. According to BSFA, technical efficiency scores of the countries were estimated and, according to these scores, rankings of efficiency were obtained. Based on the technical efficiency, while the most effective country was Australia, the lowest effective country was Turkey.

For the second step, we regress efficiency scores on a set of 20 indicators capturing health system characteristics. Unlike other similar studies, in this study, Bayesian beta regression was performed to overcome the problems of lack of degrees of freedom and model uncertainty in this regression for the benefits of robustness.

Bayesian beta regression model was constructed to obtain the related factors with efficiency scores. While regulation of prices billed by providers (*X*_6_), regulation of the workforce and equipment (*X*_8_), patient choice among providers (*X*_9_), price signals on users (*X*_11_), degree of decentralization (*X*_15_), and scope of basic coverage (*X*_19_) were related factors with the health system efficiency for Bayesian beta regression, the other variables are not related to health system efficiency scores. In particular the lowest effective countries such as Turkey, Slovak Republic, Hungary, and Italy should reconsider the health system policy and take precautions to improve the health system efficiency paying attention to six important factors mentioned and highlighted above.

## Figures and Tables

**Table 1:** Posterior mean, sample standard deviation (SD), Monte Carlo error (MC error), and median with a 95% posterior credible interval.

Node	Mean	SD	MC error	2.5%	Median	97.5%	Start	Sample
Constant	12.713	1.231	0.045	10.713	11.913	13.113	12000	37500
GDP	7.631	0.226	0.009	6.816	7.126	7.436	12000	37500
Health expenditure	1.625	0.085	0.002	1.323	1.417	1.671	12000	37500
Education	0.932	0.456	0.015	−0.045	10.021	1.524	12000	37500
Tobacco consumption	1.441	0.036	0.001	0.817	1.563	1.971	12000	37500
Alcohol consumption	0.673	0.423	0.007	−0.126	0.871	1.320	12000	37500
Fruit and vegetables consumption	1.273	0.185	0.003	0.871	1.345	1.824	12000	37500
Nitrogen oxide emissions	0.717	0.167	0.002	0.218	0.894	1.023	12000	37500
Time	1.231	0.027	0.001	0.101	1.321	1.444	12000	37500
Lambda	0.128	0.063	0.002	0.071	0.112	0.011	12000	37500

**Table 2:** Health system efficiency scores and ranking for BSFA.

Country	Efficiency scores	Rank
Australia	0.991	1
Greece	0.987	2
Korea	0.957	3
Iceland	0.955	4
Mexico	0.955	5
Switzerland	0.953	6
Sweden	0.947	7
Germany	0.944	8
Japan	0.943	9
France	0.939	10
Canada	0.935	11
Netherlands	0.935	12
Portugal	0.933	13
Austria	0.932	14
Norway	0.922	15
Belgium	0.917	16
New Zealand	0.917	17
Luxembourg	0.916	18
Poland	0.914	19
Ireland	0.911	20
Finland	0.908	21
Spain	0.905	22
Denmark	0.899	23
Czech Republic	0.895	24
United Kingdom	0.891	25
Italy	0.824	26
Hungary	0.812	27
Slovak Republic	0.808	28
Turkey	0.805	29

**Table 3:** The posterior mean, SD, and MC error with a 95% posterior credible interval.

Variables	Mean	SD	MC error	2.5%	97.5%
(Intercept)	−0.117	0.202	0.0081	−0.278	0.173
*X*_1_	0.138	0.093	0.0033	−0.041	0.244
*X*_2_	0.007	0.048	0.0019	−0.047	0.091
*X*_3_	0.011	0.053	0.0020	−0.045	0.102
*X*_4_	0.026	0.119	0.0047	−0.141	0.142
*X*_5_	−0.044	0.079	0.0033	−0.159	0.067
**X**_6_	**0.143**	**0.013**	**0.0052**	**0.026**	**0.258**
*X*_7_	−0.034	0.091	0.0031	−0.118	0.092
**X**_8_	**0.093**	**0.055**	**0.0018**	**0.021**	**0.140**
**X**_9_	**0.077**	**0.011**	**0.0004**	**0.005**	**0.228**
*X*_10_	−0.047	0.077	0.0028	−0.127	0.046
**X**_11_	**0.149**	**0.024**	**0.0011**	**0.012**	**0.253**
*X*_12_	0.077	0.181	0.0067	−0.135	0.254
*X*_13_	0.039	0.078	0.0025	−0.067	0.146
*X*_14_	0.032	0.164	0.0061	−0.100	0.271
**X**_15_	**0.112**	**0.009**	**0.0004**	**0.062**	**0.218**
*X*_16_	0.035	0.242	0.0087	−0.193	0.401
*X*_17_	0.018	0.121	0.0034	−0.152	0.124
*X*_18_	0.195	0.141	0.0029	−0.109	0.454
**X**_19_	**0.159**	**0.026**	**0.0011**	**0.028**	**0.336**
*X*_20_	−0.013	0.112	0.0039	−0.212	0.098
